# An Updated Review of Macro, Micro, and Nanostructured Hydrogels for Biomedical and Pharmaceutical Applications

**DOI:** 10.3390/pharmaceutics12100970

**Published:** 2020-10-15

**Authors:** Caroline S. A. de Lima, Tatiana S. Balogh, Justine P. R. O. Varca, Gustavo H. C. Varca, Ademar B. Lugão, Luis A. Camacho-Cruz, Emilio Bucio, Slawomir S. Kadlubowski

**Affiliations:** 1 Nuclear and Energy Research Institute, IPEN-CNEN/SP, Av. Prof. Lineu Prestes, No. 2242, Cidade Universitária, São Paulo 05508-000, Brazil; caroline.lima@usp.br (C.S.A.d.L.); tatianabalogh@hotmail.com (T.S.B.); justinepaula@usp.br (J.P.R.O.V.); ablugao@gmail.com (A.B.L.); 2 Departamento de Química de Radiaciones y Radioquímica, Instituto de Ciencias Nucleares, Universidad Nacional Autónoma de México, Circuito Exterior, Ciudad Universitaria, México CDMX 04510, Mexico; 95.luis.camacho@gmail.com (L.A.C.-C.); ebucio@nucleares.unam.mx (E.B.); 3 Institute of Applied Radiation Chemistry (IARC), Lodz University of Technology, Wroblewskiego No. 15, 93-590 Lodz, Poland; slawomir.kadlubowski@p.lodz.pl

**Keywords:** macrogel, microgel, nanogel, synthesis, biomedical applications, pharmaceutical applications

## Abstract

Hydrogels are materials with wide applications in several fields, including the biomedical and pharmaceutical industries. Their properties such as the capacity of absorbing great amounts of aqueous solutions without losing shape and mechanical properties, as well as loading drugs of different nature, including hydrophobic ones and biomolecules, give an idea of their versatility and promising demand. As they have been explored in a great number of studies for years, many routes of synthesis have been developed, especially for chemical/permanent hydrogels. In the same way, stimuli-responsive hydrogels, also known as intelligent materials, have been explored too, enhancing the regulation of properties such as targeting and drug release. By controlling the particle size, hydrogel on the micro- and nanoscale have been studied likewise and have increased, even more, the possibilities for applications of the so-called XXI century materials. In this paper, we aimed to produce an overview of the recent studies concerning methods of synthesis, biomedical, and pharmaceutical applications of macro-, micro, and nanogels.

## 1. Introduction

Hydrogels are three-dimensional networks of cross-linked polymer chains that have a high capacity to absorb water. Hydrogels have numerous applications in several areas, but in the biomedical field, they are especially helpful. Their adjustable features and unique behavior make them suitable to produce different types of materials such as contact lenses, blood-contacting hydrogels, scaffolds, wound-healing bioadhesives, artificial kidney membranes, artificial skin, vocal cord replacement, and artificial tendons. This wide application variability and their relevance make them materials of great interest in research [1]. Concerning pharmaceutical applications, the presence of the pores in the hydrogel structure is very attractive for drug delivery systems considering that they can carry active ingredients and release them at a controlled rate by the swelling of the semisolid formulation or even by its disintegration, among other mechanisms.

The possibility to design a hydrogel according to the desired application requirements is related to the wide range of polymers that can be obtained. Hence, it is possible to control their biodegradation rate, mechanical strength, swelling capability, and responsiveness to external stimuli by combining natural and/or synthetic polymers [2].

Since hydrogels must be crosslinked in their nature, the crosslinking process is of special importance when designing these systems. This cross-linking process may be carried out by radical reactions, by mixing monomers and oligomers to form the 3D structure or, as an alternative, initiated with the polymer chains that may react with each other by using ionizing radiation or UV light to excite functional groups within the molecules so they may react with each other. When carrying out the crosslinking procedure, chemical cross-linkers (molecules that may contain two or more functional groups that may participate in polymerization reactions) is another option to obtain the network structure [1]. It is important to mention that by using these methods chemically cross-linked hydrogels are provided; however, it is also possible to obtain physical ones by intermolecular interactions such as ionic, hydrophobic, and hydrogen bonds [3].

**Figure 1:**
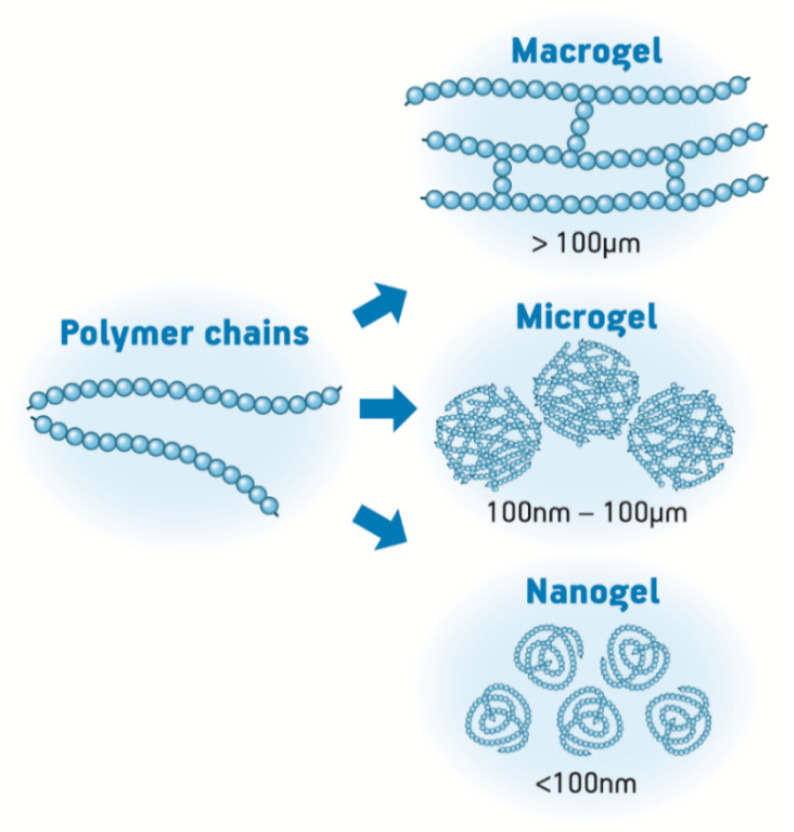
Representative scheme of gels at different size levels.

Depending on the size of the obtained particles, hydrogels may be classified as macro-, micro- or nanogels. When they are smaller than 100 nm, they are usually considered nanogels; while, gels with particle sizes bigger than this (up to the micrometer range) are called microgels. Finally, if these gels have particle sizes bigger than 100 µm, they are usually called macrogels (Figure 1). Differently from the bulk gels, micro- and nanostructures behave as macromolecules that rapidly respond to external stimuli, which makes them functional or smart materials. Additional to these hydrogel architectures, hydrogel substrates may be attached to other substrates as grafts, to add functionality to these substrates and increase the versatility of hydrogels [4]. The particular performance of these systems is a consequence of their unique architecture, which is dynamic, permeable, and deformable [3,5]. These features are of high interest to increase stability when carrying biopharmaceuticals such as proteins [6].

Hydrogels may also be combined with other materials on the nanoscale, giving rise to nanocomposites with different characteristics such as antibacterial activity, high mechanical strength, and magnetic properties [7]. Zhang and coworkers [8], for instance, developed a chitosan hydrogel containing Fe_3_O_4_ magnetic nanoparticles for the delivery of immunotherapy for bladder cancer treatment.

Although many studies have already been published regarding these materials, the increasing use of hydrogels to solve different challenges continues to position them as an ongoing research subject. Thus, in this work, we aimed to produce an updated overview of hydrogels, exploring the peculiarities of methods of synthesis and applications in the biomedical and pharmaceutical field of their bulk-, micro-, and nanosize levels.

## 2. Macrogels

### 2.1. Definition

Hydrogels (also called macrogels) are a specific class of polymeric materials. Dorothy Jordan Lloyd said that “the colloidal condition, the gel, is one which is easier to recognize than to define” [9]. At this moment hydrogels are defined as systems of at least two components consisting of a three-dimensional network of polymer chains and water that fills the space between the macromolecules [10]. Based on the chemical nature of substrates used for synthesis, as well as on the structure and density of the network, hydrogels in equilibrium can hold various amounts of solvent. That is why, in their swollen state, the amount of water is much higher than the dry mass of the polymer.

Two main groups of hydrogels can be distinct: (a) physical gels (pseudo gels), with the chains linked by secondary forces, e.g., hydrogen bonds, electrostatic forces, chain entanglements, or hydrophobic interactions and (b) chemical (true, permanent) hydrogels with covalent cross-links.

In order to hold, at equilibrium, an excess of water, polymers used in these materials are usually of moderately hydrophilic character e.g., poly(ethylene oxide) (PEO), poly(vinyl alcohol) (PVAL), poly(N-vinylpyrrolidone) (PVP), and poly(2-hydroxyethyl methacrylate) (PHEMA). Besides that, hydrogels for biomedical applications are obtained from natural polymers, especially polysaccharides.

**Figure 2:**
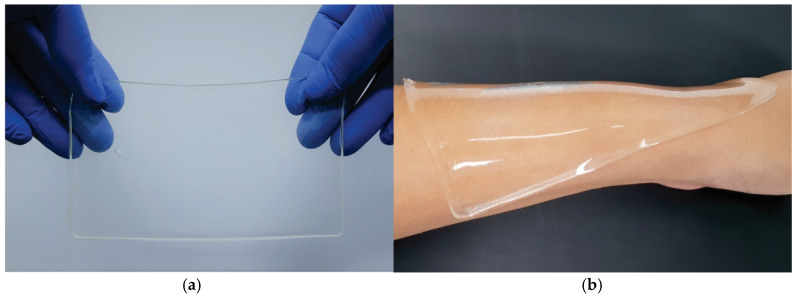
Chemically crosslinked hydrogel crosslinked and sterilized simultaneously using gamma radiation; (**a**) hydrogel dressing and (**b**) hydrogel dressing being used in human skin.

Among the most important, successful, and promising application of hydrogels is their use in medicine and pharmacy (soft contact lenses, wound dressings, drug-delivery systems, superabsorbents, etc.) [11,12,13,14,15,16,17,18,19,20,21,22,23,24,25,26,27,28]; however, several non-biomedical applications (e.g., in agriculture, environment protection, sanitary) have been also utilized. An example of such applications (hydrogel-based wound dressing) is shown in Figure 2.

For four decades, a specific class of hydrogels, capable of reacting to various environmental stimuli such as temperature, pH, ionic strength, solute concentration, electric field, light, sound, etc., is tested for use in the so-called ”intelligent biomaterials” [29,30,31,32,33,34]. Certainly, there is still a big gap between the artificial hydrogel fish that moves by swinging its tail in a laboratory bath [35] and the implementation of this development in practical applications such as artificial muscles. However, rapid progress in this field, correlated with increasing demands for more effective medical treatment indicates that there is no exaggeration in including these systems in the list of the “materials of the XXI century”.

### 2.2. Methods of Synthesis

Hydrogels may be obtained by a number of chemical methods. These may comprise one-step procedures such as simultaneous polymerization and crosslinking of multifunctional monomers, as well as multiple-step techniques based on the synthesis of macromolecules with reactive groups and their subsequent crosslinking or on the reaction of the polymers with suitable crosslinking agents [36].

To understand the various chemical methods for the production of hydrogels, it is important to make a differentiation between some classical methods for producing polymers and how the application of these methods may affect the characteristics of a hydrogel. The first procedure that we will describe is bulk polymerization i.e., polymerization in the absence of an added solvent.

Examples for a homogenous bulk polymerization are methyl methacrylate and styrene. Bulk polymerization is more often used for step-growth polymers such as polyurethanes, polycaprolactam (Nylon 6), polycarbonates, and polyester (PET). In the case of hydrophilic polymers, homogenous, non-porous poly(N-vinylpyrrolidone)-based hydrogels are also obtained with this method. These glassy, transparent polymer matrixes are very hard until immersed in water, when the network, up to some degree, swells and becomes soft and flexible. Homogeneous hydrogels such as these have been used widely in various applications, especially in the controlled drug delivery area where limited diffusional characteristics are required.

Hydrogels (e.g., poly(acrylic acid), poly(N-vinylpyrrolidone) can also be prepared by solution polymerization and subsequent crosslinking. This procedure consists of polymerizing monomers in a solvent, which is good both for the monomers and the polymer chains, namely, a solvent that allows for the solubility of both the monomer and polymer substrates. The crosslink density of the product depends on the nature of the monomer, the amount of diluent in the monomer mixture and the quantity of crosslinking agent and/or delivered energy. Hydrogels with effective pore sizes in the 10–100 nm range and in the 100–10 µm range are termed “microporous” and “macroporous” hydrogels, respectively.

With this polymerization technique, porous hydrogels can also be obtained by addition of the porogen (micronized sucrose, lactose, and dextrin, sodium chloride, and poly(ethylene glycols)) which may later be removed by washing the final product with water to leave an interconnected meshwork [37].

As discussed previously, in solution polymerization, the solvent must be compatible with both the monomer (or monomers) and the resulting polymer. When the diluent is a non-solvent for the formed polymer (e.g., PHEMA in water), the solubility of formed polymers decreases dramatically with the polymerization progress. Polymer-rich monomer droplets are being formed that join together, and a network filled with large spaces (i.e., heterogeneous, porous hydrogels) is obtained by the end of the polymerization process. Such process is called a heterogeneous solution polymerization or dispersion polymerization [38,39].

To obtain porous hydrogels, one can also alter solvent “quality” by removing a good solvent or adding a non-solvent to a polymer solution. It can be also performed by changing the reaction temperature which e.g., for gelatin causes gel formation below the critical miscibility temperature and for rapidly frozen solutions subsequent removal of water by freeze-drying sublimation. In the case of polymers with a lower critical solution temperature (LCST), phase separation occurs as the temperature is increased above this value. Porous hydrogels of poly(N-isopropyl acrylamide) (PNIPAM) and poly(vinyl methyl ether) (PVME) have been obtained with this method. The other idea is to surface-crosslink individual hydrogel particles to form crosslinked aggregates of particles with pores between them [40].

Considering hydrogel synthesis for medical purposes, even though these procedures allow obtaining products of anticipated properties, they have substantial disadvantages since such materials should not contain any usually toxic residues of monomers, initiators, crosslinking agents, or additives. They have to be removed from the product in the other time-consuming, technological steps. This complicates the implementation of this technology and may lead to a significant increase in production costs.

Ionizing radiation has been since long recognized as a very suitable tool for the formation of hydrogels (e.g., Rosiak) [10,41]. Easy process control; the possibility of combining hydrogel formation and sterilization in one technological step; no necessity to add any initiators, crosslinkers, etc.; no generation of waste; and relatively low running costs are all reasons that make irradiation one of the most important methods of choice for the synthesis of hydrogels, especially those designed for biomedical applications. However, this technique also has some limitations, and not all polymers can be used to obtain hydrogels by this procedure. The main polymers used to obtain polymeric networks are poly(ethylene oxide), poly(vinyl alcohol), and poly(N-vinylpyrrolidone).

The first experiments with radiation crosslinking of hydrophilic polymers started in the early fifties by pioneers of the radiation chemistry of polymers Charlesby [42] and Chapiro [43]. At that time, researchers mainly focused on mechanisms of radical formations, their reaction leading to the formation of a network with different topologies as well as the influence of irradiation properties on that process. In the late sixties, papers and patents, mainly from Japanese and American scientists (Takasaki Radiation Chemistry Research Establishment headed by Kaetsu as well as Hoffman and his colleagues from the Center of Bioengineering, University of Washington), started a new era of hydrogel-based biomaterials formed using a radiation technique. The first data were mainly connected with immobilization of biologically active species in hydrogel networks, new drug delivery systems, and enzyme traps as well as modification of material surfaces to improve biocompatibility and ability to bond antigens and antibodies [12,44].

### 2.3. Biomedical Applications

Almost since the beginning, besides synthetic procedures and determination of physicochemical properties of the hydrogels, the work on hydrogels has been also dedicated to their applications. Starting on the early work [45] on the synthesis of PHEMA hydrogels and their use as contact lenses up to novel drug delivery systems [19,25], the work has focused on new material synthesis or modification as well as evaluation and possible market analysis.

Between different possible fields of applications, the biomedical field seems to be the most frequently considered with several products already successfully transferred to the market. This is mainly connected to the fact that the most important properties of hydrogels, i.e., ability to swell without losing their shape and mechanical strength, are commonly met in a number of natural elements of a human body (muscles, tendons, cartilage, etc.). The other important issue is good biocompatibility in contact with blood, bodily fluids, and tissues.

Wound dressings are of the most successful examples of the commercialized hydrogel-based materials. Rosiak and coworkers have presented a successful methodology of hydrogel dressing production based on high-energy radiation [10]. The hydrogel dressings obtained with the proposed methodology provide several desirable properties: (a) maintain a moist environment, (b) provide a barrier to bacterial contamination while (c) allowing oxygen access to the injured area, (d) can be removed without damage to the healing surface. The most important reported fact was an increased healing rate compared with dry gauze dressing [46]. Despite these advantages, the most significant technological issue in the presented method is the connection between synthesis and sterilization in a single step. In a simplified way, ionizing radiation interacts with polymers forming radicals that may interact mutually with host molecules or with the surrounding medium. Covalent bonds may be formed by radicals present in different polymer chains, and when there are enough of these bounds, the hydrogel is synthesized [10]. On the other hand, in 1995, sterilization by radiation was introduced as an International Guideline in 1995 for biomedical products. Basically, there are two approaches: one that recommends a minimum dose of 25 kGy to guarantee sterilization, and another that guides to find a specific dose to a certain product [47].

An important and novel achievement of practical use of hydrogels is their application in tissue engineering, thus many excellent reviews have been published (e.g., Hoffman [19,25]) in this topic. In general, both advantages (aqueous environment protects cells and fragile drugs, good transport of nutrient to cells and products from cells, easy modification with cell adhesion ligands, the possibility of injection in vivo as a liquid that gels at body temperature, biocompatibility) and disadvantages (hard to handle, usually mechanically weak, difficult to load drugs and cells and then crosslink in vitro as a prefabricated matrix, difficult to sterilize) are often present when dealing with examples of natural and synthetic polymers (or their combinations) used for synthesis; therefore, the opportunity to improve current hydrogel implementations is still very promising.

### 2.4. Pharmaceutical Applications

The great ability of most of the hydrogels of absorbing large amounts of water makes them immunotolerant materials which tend to be very attractive for biological applications. Their flexibility is also another feature that makes them very similar to natural tissue which is important to avoid potential irritations or other immune responses. Apart from these properties, it is also possible to work with the balance between hydrophilic and hydrophobic areas of the gel, in accordance with the solvent diffusion characteristics wanted in a way that is possible, for example, to control the release of a drug. By using this alternative, drug delivery has become one of the main applications of hydrogels in the pharmaceutical field [48]. “Smart” or “intelligent” hydrogels are the most studied variety in the development of drug delivery systems to carry both low molecular weight drugs to macromolecular ones, even examples featuring insulin and other peptides. The main interest in this area of research is to protect the active molecules and to release them via diffusion or erosion [25,49,50].

Mandru and coworkers [51] developed a system of poly(vinyl alcohol) and a thermoreversible polyurethane by a freezing-thawing method for drug delivery applications. The researchers showed that the physical hydrogel prepared presented pore sizes between 4.05 and 39.05 µm and the capacity of water swelling up to 1675%. The drug delivery investigation was carried out with neomycin sulfate, and it was observed that its release was following the swelling behavior of the hydrogel. The results demonstrated that in about 42 min, a release plateau was achieved, and by this time, all the chains in the matrix attained high mobility because of the material swelling, allowing the release of the drug.

In cancer treatment, hydrogels may be used for different therapies. In chemotherapy, they are used to reduce side effects, direct the drug to the target site, and improve its therapeutic potential and even avoid metastasis. For instance, nanocomposite hydrogels are applied in hyperthermia and radiation therapy, most of them with stimuli-responsive features. pH-sensitive systems are among the most studied for cancer therapy because of their fast structure transitions against changes in the pH environment [52].

Hydrogels may also be a tool to carry hydrophobic drugs such as Cyclosporine A (CsA). CsA is a high molecular weight immunosuppressive drug usually applied to treat diseases such as rheumatoid arthritis and to prevent rejection in patients that have been through organ transplants. However, its intravenous or oral administration comes along with several side effects such as vomiting, confusion, fever, kidney, and liver dysfunction. Considering all that, researchers developed an injectable and in situ forming hydrogel made of a hyaluronic acid-calcium complex and sodium alginate to deliver CsA. Characterization assays showed that the gelation time of the system could be controlled varying the range between the two polymers in a period from 2 to 45 min, and that the interconnected pores with diameters from 20–300 µm were suitable to carry macromolecular drugs such as CsA. Their system presented a sustained in vitro release of up to 14 days following the biodegradability of the hydrogel. The researchers also investigated if the hydrogel effluents could cause any effects on blood coagulation and demonstrated that they represented no threat, demonstrating its high potential for this application [53].

## 3. Microgels

### 3.1. Definition

Microgels are polymer chains intramolecularly crosslinked in small dimensions—from hundreds of nanometers to some micrometers—dispersed in colloidal solutions. Their structure is very close to solid particles, once their surface is well established [54,55]. These kinds of gels have a high capacity for water content, large surface area, and an interior network useful for drug delivery systems. Biopolymer-based microgels are of great interest for drug delivery and tissue engineering systems because of their properties such as biodegradability, nontoxicity, and relatively low cost, beyond being abundant in nature. Additionally, these materials normally contain a high density of functional groups, namely hydroxyl, amino, and carboxylic acids, which are helpful, for example, for bioconjugation with cell-targeting agents [56].

Colloids may be classified as rigid particles, flexible macromolecules, or micellar aggregates based on surfactants; however, microgels do not fit into only one of these definitions once they are macromolecular networks swollen by a solvent. The presence of the solvent in the microgels makes them soft materials. In other words, they can have soft interaction potential and are deformable without losing their structural integrity. The way microgels interact with their surroundings is different from other colloidal systems; this happens because they tend to exchange solvent and solutes with the environment causing changes in its size and shape. That means that some microgels may be sensitive and responsive to the environment they are placed in [57]. High water absorption capacity is a property often presented by microgels mainly because most of them have crosslinked polymer chains that tend to be hydrophilic and absorb water instead of dissolve in it. Their swelling property is reversible according to external stimuli which can be a change in temperature, pH, ionic strength, and solvent [58]. Furthermore, microgels present a large surface area and can incorporate biorelated molecules due to their interior network, which makes them interesting for drug delivery systems and other biomedical applications [59].

### 3.2. Methods of Synthesis

**Figure 3:**
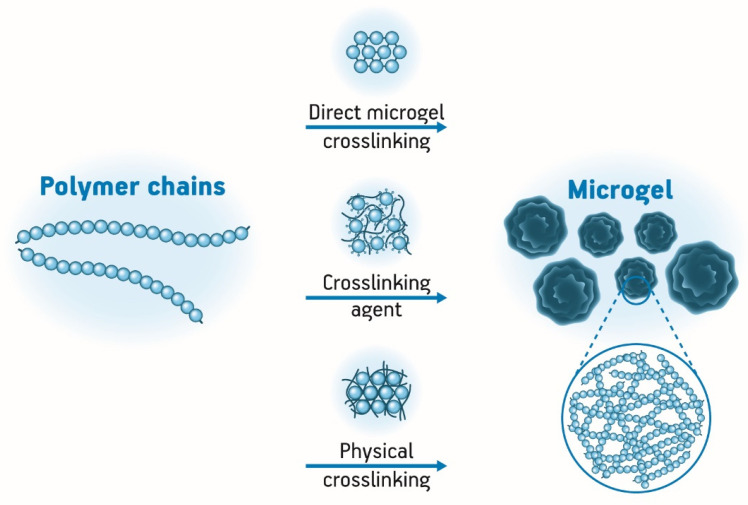
Methods of synthesis of microgels.

Essentially, microgels may be prepared by physical or chemical crosslinking of hydrophilic polymers (Figure 3). Physical crosslinking is reversible upon external stimuli once it involves non-covalent attractive forces such as ionic and hydrophobic interactions. Biodegradable physically crosslinked microgels can encapsulate drugs, cells, and proteins and release them by their degradation process. On the other hand, chemically crosslinked microgels are made of covalent bindings, which make them more stable with a permanent structure. Usually, the presence of crosslinkers is necessary to synthesize this kind of microgel [19,60,61]. Additionally, in terms of microgels synthesis, it is important to control particle size distribution, functional groups distribution, and colloidal stability [61,62].

There are several routes to produce microgels which can be divided into homogeneous nucleation and polymerization, emulsification, or complexation methods. Basically, in the first case, the microgel is obtained from homogeneous solutions; in the second, aqueous droplets are dispersed in an oil phase followed by crosslinking; and, in the last, two water-soluble polymers are put together to form complexes with each other [63].

Homogeneous nucleation and polymerization methods are important to describe because of their relevance. These methods consist in mixing water-soluble monomers with crosslinking agents and an initiator. This type of process can be performed by emulsion polymerization using a water-soluble monomer, a radical initiator, and surfactants in an aqueous medium. To produce core-shell microgels, this emulsion step is followed by a second polymerization to form the shell. Microgels can also be made by water-in-oil heterogeneous emulsifications from the combination of a continuous oil phase, with oil-soluble surfactants and droplet emulsions of water-soluble polymers. After mixing all these reagents, crosslinking agents are added. The crosslinkers may be difunctional or multifunctional influencing the structures of the microgels. Several methods can be used, such as precipitation, inverse (mini) emulsion, inverse microemulsion, dispersion polymerization, membrane emulsification, or heterogeneous controlled/living radical polymerization [64]. Each one of these methods is briefly explained in Table 1.

To prepare microgels from polymer complexation methods, solutions of opposite-charged polyelectrolytes are mixed to form a complex. In this case, the polymer network will be formed by the electrostatic attraction forces between the chains, resulting in a physically crosslinked microgel [67]. Polymer complexation can also be obtained via hydrogen bonds. Several parameters such as hydrophobic effects, molar mass, and structure of the polymers, solvent, and pH influence the formation and stabilization of polymer complexes [70].

### 3.3. Biomedical Applications

**Figure 4:**
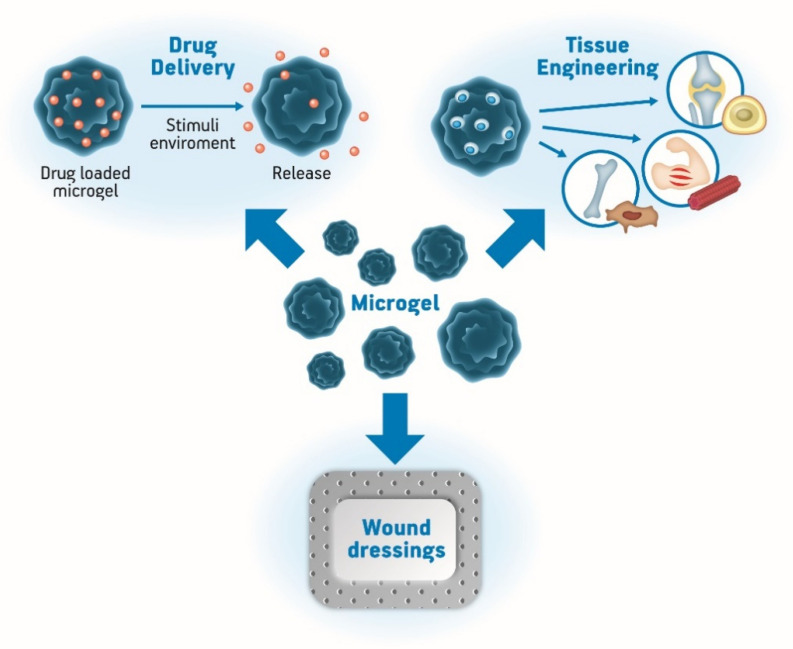
Biomedical applications of microgels.

The pursuit of new biomaterials to improve the quality of life and health of humans is a great area of research that has been gaining prominence [71]. As mentioned before, hydrogels are widely applied in the medical field because they have high water content, porosity, and soft consistency which make it possible for them to simulate natural living tissue (Figure 4), and microgels are not an exception. Furthermore, they may be stable, degrade, or even dissolve according to the purpose of the application that may be as wound dressings, tissue engineering, contact lenses, drug delivery systems, and others [28,72].

Among the several applications of microgels, delivery of chemotherapy for cancer treatment is one of great research interest. Although chemo treatments are effective in treating tumors, they have many limitations such as low specificity which is the main reason for their toxicity [73]. Because of that, polymer-drug delivery systems have been developed so that the delivery may be more specifically targeted. Among the possible solutions to target tumor sites and reduce side effects is by using a pH-responsive system. Eswaramma et al. [74] designed novel pH-sensitive interpenetrating polymer network microgels by combining chitosan and modified guar gum for controlled release of anticancer agent 5-FU. Scanning Electron Microscopy analysis showed that the microgel diameter was about 130 µm. In vitro drug delivery assays were performed, and 98% of the chemotherapeutic was released in 48 h when in an acid environment as in the vicinity of a tumor, where the pH was around 1.2. This slow and pH-dependent release of the drug suggests that this microgel might be appropriate for cancer targeting [74].

In the tissue engineering field, osteogenic bone mesenchymal stem cells are applied to repair bone injuries; however, low cell retention is still a disadvantage in this technique [75]. A possible solution for that is to use polymeric scaffolds to deliver those cells and increase the retention in the tissue, helping the regeneration process. Thus, microgels have been the focus of studies for cell delivery once they can be injected, solidified in situ, and carry not only stem cells but also growth factors. In addition to the high surface area that is important to transfer nutrients, microgels can escape phagocytosis so they can remain in the injury site. Hou et al. [76] developed degradable PVA microgels for osteogenic bone mesenchymal stem cells and growth factor encapsulation. The biomaterial was produced by using a microfluidic flow-focusing device and presented suitable elasticity, degradation, and bioactivity. The authors observed that the low rate of crosslinking with the biocompatible polymers was crucial to ensure prolonged survival, proliferation, and migration of the cells. Additionally, the slow release of the growth factor by PVA reinforced osteogenic differentiation in vitro, highlighting the great potential of the microgel for application in regenerative medicine [76].

Busatto et al. [77] designed a microgel made of hyaluronic acid to encapsulate hydrophobic drugs. Hyaluronic acid is the main constituent of the extracellular matrix in the human body and has many interesting properties such as biocompatibility and biodegradability. The release of the active agent present in a hyaluronic acid matrix happens by the enzymatic erosion of the polymer. It is known that the hyaluronidase is strongly expressed in several carcinomas, and, because of that, hyaluronic acid has been used to deliver chemotherapeutics. Therefore, in this research, the authors studied how to prepare a system capable of encapsulating hydrophobic drugs. To do that, they used a hyaluronic acid modified with methacrylate groups in an oil-in-water emulsion method followed by photopolymerization to create cross-linkages. Degradability and release assays showed that the microgel was efficient.

Microgels may also be used in the biomedical field to produce wound dressings. Wilke et al. [78] produced biocompatible microfibers loaded with nano-ZnO nanoparticles from poly(ε-caprolactone) (PCL) microgel with carboxylic groups in its core. Zinc oxide is an antioxidant and has antibacterial properties that make it valuable for wound treatment. To prepare the fibers, first, a microgel carrying carboxy-groups was made and nanoparticles of zinc oxide were grown by a precipitation process in its pores. After that, linear PCL chains were applied as building blocks with the microgel containing the nanoparticles to electrospun the fibers. The studies demonstrated that the fibers were biocompatible, were capable of releasing zinc ions for five days, and had the potential for application in medical devices.

Microgels are materials of great interest in the biomedical field because they represent a possible solution for limitations of macrogels performance in some applications, for example, in drug delivery systems. Their properties such as the high capacity of water uptake, controlled drug release, large superficial area, and possibility to respond to stimuli make them extremely versatile materials with a wide range of uses for new research. 

### 3.4. Pharmaceutical Applications

Microgels have important properties that make them very attractive to the pharmaceutical field. Their lower viscosity, higher surface area, and rapid response to stimuli in physiological conditions make them even more interesting than hydrogels. In this way, they represent a solution for rapidly metabolized and protect sensitive drugs [79].

Biomacromolecular drugs, for instance, need such systems to provide conformational stabilization, protect them from degradation, and control their release rate, which causes the reduction in toxicity, thus preventing side-effects [80]. Besides that, molecules such as growth factors, hormones, enzymes, and antibodies have short half-lives [81]. The highly hydrophilic nature of most hydrogels is responsible for limiting aggregation and conformational changes of the biomacromolecules. This is important to maintain their biological effects. As microgels have the capacity to behave in a responsive way, according to pH, ionic strength, temperature, external fields, and other changes in the environment, they are very suitable to carry and release biomacromolecules such as protein and peptides [82].

Jooybar and coworkers developed a biodegradable microgel system based on hyaluronic acid and tyramine for encapsulation of proteins such as lysozyme and TGF-β1 growth factor. The system was prepared in an inverse microemulsion and crosslinked by and enzyme. In this study, the researchers demonstrated that the drug delivery system could maintain a sustained release for more than four weeks through diffusion and degradation of the microgel [81].

Another improvement that microgels can offer to the delivery of cells and proteins is the possibility of treating damaged tissues administrating them via catheters or injections with needles of reduced diameters, avoiding more complicated procedures such as surgery to implant other biomaterials. Foster et al. [83] developed synthetic microgels of poly(ethylene glycol) with controlled size for the triggered release of proteins. In their work, the gel network was crosslinked with a protease degradable peptide that allowed the release of the proteins covalently bonded to the microgel. The studies demonstrated that the microgel injected into mice subcutaneously stimulated the vascularization by releasing vascular endothelial growth factor and promoted tissue regeneration after microgel degradation.

Microgels can also be used in the pharmaceutical field as biosensors, which are devices capable of detecting and quantifying biological molecules. Among the most famous examples of this application is the sensor for glucose, used for patients facing diabetes mellitus, which is considered a serious global health threat. A person who leaves with diabetes demands continuous glycemic control. Usually, these patients are treated with palliative self-injections of insulin that are not accurate according to their daily glycemic levels and impair their quality of life [5,84,85]. Matsumoto and colleagues developed a smart copolymer microgel system that responds to the presence of glucose such as an artificial pancreas. Their microgel was based on radical copolymerization of 2,2′-azobisisobutyronitrile (AIBN) as an initiator in the presence of N,N′-methylene-bis(acrylamide) (MBAAm), and the results presented a glucose-induced phase transition system, able to manage the release of insulin [85].

The treatment of osteoarthritis with microgels is a current example of a pharmaceutical application of these materials as biological lubricants. The degeneration of the articular cartilage and the synovial fluid due to age and other factors raises the need for alternative treatments since the self-repair of cartilage is a very difficult process owed to the low quantity of blood vessels in it. Microgels stand out in this application when compared to macrogels because of their higher mechanical properties combined with good lubrication and rheology, besides the capacity of loading drugs and stimuli responsiveness [84]. Biolubricants also may be used in the eyes, oral cavity, and gastrointestinal tract, among other applications in order to minimize discomfort. A recent study presented a starch-based emulsion microgel containing oil. The samples had their lubrication performance tested under physiological conditions to skin and oral cavity mimicking conditions. The encapsulated oiled were proved to be released under tribological shear and enzyme activity promoting lubrication. The model developed also showed potential for the targeted and controlled delivery of lipophilic nutrients and pharmaceuticals [86].

In fact, drug delivery systems containing microgels can be explored in several ways. Mahdieh and Holian [87] in their recent work showed electruspun fibers loaded with poly(n-isopropylacrylamide) microgel particles with potential for future applications concerning the release of therapeutic agents. Their microgel was developed using a novel ball-milling method, as a non-toxic alternative to produce the particles which were loaded in the core of the fibers together with silver nanoparticles, used as a model antibacterial drug. Other advantages and possibilities of using microgels for drug delivery are promoting bioavailability and engineering multidrug and on-demand release systems [5].

Although many studies with novel applications of smart microgels are available, industrial application is still in the primary stage because of the lack of clinical data concerning safety and effectiveness in vivo. Therefore, this kind of study may be included in future research to establish materials with prolonged controlled drug released and assured safety. Improvements concerning encapsulation of insulin, for example, and more proof of the concept of microgels as biolubricants are still necessary. Integrated systems of diagnosis and treatment are also future trends for microgel applications once there is some investigation in this area [84].

## 4. Nanogels

### 4.1. Definition

Nanogels are innovative systems on the nanometer-scale of great potential in nanomedicine, pharmaceutics, and bionanotechnology. Their internal structure is similar to that of hydrogels or microgels; however, there is variation in size (up to 100 nm) and responsiveness leading to several advantages such as the capacity of injection into the circulation reaching target tissues and the ability to deliver their payloads locally and intracellularly. Furthermore, the nanoscale size improves the solubility of hydrophobic drugs, increases drug accumulation in tumors, enhances the stability of therapeutic agents against enzymatic and chemical degradation, and decreases cytotoxic side effects. The nanogels present a high potential of drug encapsulation, large surface area, and stable interior network structure [88,89,90,91,92].

Nanogels are defined as a two-component system on the nanometer scale consisting of a permanent three-dimensional network of linked polymer chains, and molecules of a solvent filling the pores of this network. The particle size range of nanogels varies from 0 to 100 nm and the appearance thereof shows a coiled state of macromolecules that in contrast with the precursor linear polymers present new properties due to covalent crosslinking. Nanogels obtained from hydrophilic polymers exhibit high water content and swelling by maintaining their structure intact without dissolving. This water absorption capacity occurs due to the presence of hydrophilic functional groups such as –OH, –CONH–, –CONH_2_–, –COOH, and –SO_3_H. The hydrophilicity of nanogels contributes to some of its characteristics such as biocompatibility and high carrying capacity of hydrophilic biotherapeutics. The high-water content relates to the transport properties of biologically active molecules smaller than the pore size of the gel. Additionally, nanogels exhibit high stability and biodegradability [89,90,91,92,93,94,95].

Chemical or physical crosslinking of polymers with hydrophilic or amphiphilic macromolecular chains forms the three-dimensional structure of the nanogels. Additional functionality may be introduced by chemically modifying in formed nanogels or using carrier polymers of specific functionalities in the preparation of the material. The introduction of functional groups allows nanogels to become sensitive to stimuli such as pH, temperature, electromagnetic field, and light—an important fact in the controlled release of actives at target sites [89,91,92,95].

**Figure 5:**
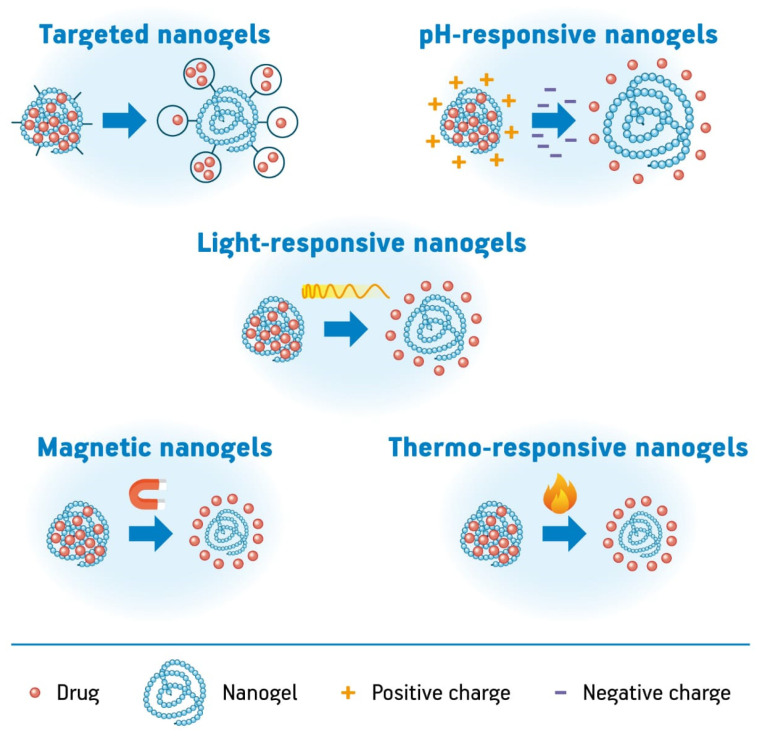
Representative scheme of stimuli-responsive nanogels.

The nanogels can be classified according to the responsive behavior as shown in Figure 5 in thermo-responsive, pH-responsive, light-responsive, magnetic nanogels, and targeted nanogels. These nanogels promote the controlled release of drugs at a specific site of action under environmental triggers. Thermo-responsive nanogels enable the release of drugs in response to temperature stimuli and can be used in the treatment of cancer. For example, nanogels prepared with PNIPAM present a volume phase transition temperature (VPTT) of 32 °C and are widely studied. In another example, nanogels based on chitosan and poly(N-vinyl caprolactam) (PVCL) demonstrated controlled release above 38 °C (their volume phase transition temperature) [94,96,97].

The pH-responsive nanogels are composed of acidic or basic groups that promote swelling–deswelling behavior depending on the pH. The slightly acidic microenvironment of tumors, endosomes, and lysosomes provides the release of drugs from pH-responsive nanogels. pH-sensitive polymers such as poly(2-(diethylamino)ethyl methacrylate) (PDEAEMA) are used in the preparation of these nanogels allowing the sustainable release of the cargo molecules due to their pH-responsiveness at physiological conditions. There are many studies involving triggers controlled exogenously for the swelling–deswelling behavior of the nanogels. This control can be exercised by irradiation with light or exposure to electrical and magnetic fields [94,98,99,100]. 

Another kind of nanogels are those which are stimulated by light to promote the controlled release of active molecules. The wavelength, light intensity, and duration of exposure are parameters used to obtain the desired therapeutic effect. Gold nanoshells, gold nanorods, and gold nanocages are examples of photosensitive nanoparticles applied in photothermal therapy. Nanoparticle-based nanogels have been demonstrated as a promising emerging candidate for drug delivery. Besides these structures, nanogels fabricated from light-responsive polymers have also been studied for this functionality.

Magnetic nanogels have been studied for application as nanocarriers of drug and genes, as well as for applications on magnetic resonance imaging (MRI). These structures present the ability to respond in the presence of a magnetic field based on superparamagnetism which is a form of magnetism where superparamagnetic materials are attracted by an external magnetic field [94,98,99,100].

Targeted nanogels are structures functionalized with specific targeting groups such as hormones, nucleic acids, ligands, peptides, and receptors which allow the release of actives in the target cells, organs, or tissues. These groups anchor or bind the nanogels to the target site improving the internalization of the nanogels and drug delivery and avoiding adverse effects [94,101].

### 4.2. Methods of Synthesis

Different synthetic routes have been used for the development of nanogels that can be classified into two main categories: chemically crosslinked nanogels and physically crosslinked nanogels, according to their crosslinked structure. The chemically-cross-linked nanogels present covalent bonds linking the polymer network and making them stable, rigid, and permanent, while the physically crosslinked nanogels present non-covalent bonds, which are weaker linkages, thus allowing sol-gel phase transitions as a result of the environmental stimuli [92,93,102].

Currently, several methods have been developed to obtain nanogels with a significant characteristic for biomedical application that is the stability of the gel particles in dispersion. This stability is influenced by the control of the particle size, crosslinking type of the polymer chains, and nature and chemical composition of the polymer matrix. In this way, the chemically crosslinked nanogels are more interesting due to the size stability and reproducibility.

The main methods of synthesis of nanogels are divided into two groups, one of which is known as crosslinking polymerization and involves techniques based on simultaneous polymerization and crosslinking, using monomers or their mixtures as substrates. The other group covers methods based on the crosslinking of macromolecules from polymer precursors which are polymers such as amphiphilic or triblock copolymers capable of forming nanogels by self-assembly or polymers with many reactive sites which can be directly used for chemical crosslinking.

Apart from these two groups, nanogels can also be prepared by controlled aggregation by physical self-assembly of hydrophilic polymers and template-assisted fabrication of nanogel particles. The first method is a simple, green, and low-cost process conducted in dilute aqueous media that implies controlled association of hydrophilic or amphiphilic polymers linked by hydrogen bonds, van der Waals forces, hydrophobic forces, and/or electrostatic interactions. The second method involving photolithography or micromolding techniques, photolithography, uses exposure to ultraviolet (UV) radiation of UV cross-linkable polymers with direct collect of fabricated particles by the dissolution of the substrate in water. The micromolding techniques that are similar to photolithography, however, allow the reduction in cost since they do not use lithographic equipment. Modern methods for nanogel synthesis have been developed as shown in Table 2 that lists, in addition to recent methods, the traditional methodologies of nanogel synthesis [90,91,92,93,103,104,105,106].

The choice of the method of preparation of a nanogel must be performed carefully due to the adverse effects of the use of organic solvents and surfactants. One should also pay attention to the fact that the presence of monomer or surfactant impurities may complicate the purification process. The crosslinking of polymer precursors provides excellent properties relevant to many applications, especially when using ionizing radiation for crosslinking. It is known that the reaction of intramolecular crosslinking can be obtained by using water-soluble polymers in dilute solutions and a cross-linker capable of reacting with the chain’s functional groups.

The ionizing radiation is an alternative method of intramolecular crosslinking initiation which avoids the addition of any additives, allowing the reaction to be carried out in a pure polymer-solvent system, and in this way, one can produce nanogels for biomedical applications free from monomers, crosslinking agents, or surfactants, eliminating the purification step [90,93]. Fast electron beam radiation and gamma radiation by ^60^Co sources have been employed in the evaluation of the influence of dose rate on the competition between inter- and intramolecular recombination, allowing the formation of microgels and nanogels, respectively. Additionally, the use of ionizing radiation allows easy control of the production process of the nanogels and the possibility of adapting them to possess specific physical and chemical characteristics. However, processes involving high energy radiation are not easily implemented in existing production lines, limiting their applicability on a large scale, which is their greatest disadvantage [93,95,102,107,108].

### 4.3. Biomedical Applications

**Figure 6:**
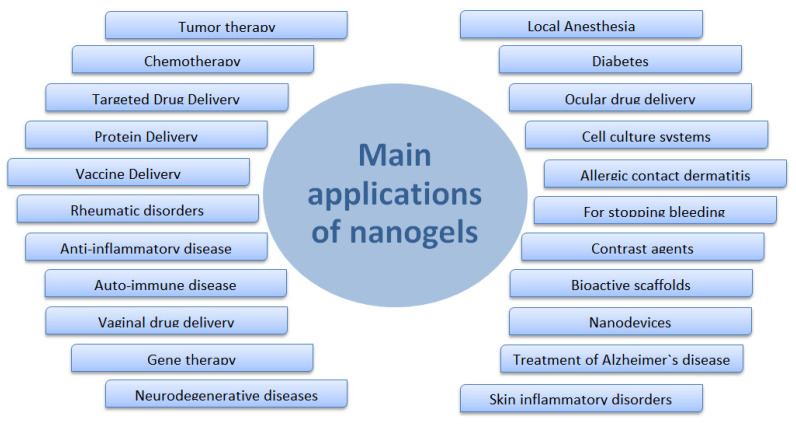
Main applications of nanogels.

Nanogels are promising and innovative materials in the field of biomedical applications. Since the emergence of this class of materials has been explored, its application as biocompatible carriers in the biotechnological and biomedical areas has been tested, with a special interest in the release of drugs due to high carrying capacity, stability, uniformity, adjustable size, ease of preparation, minimal toxicity, responsiveness to stimuli, the extensive surface area for bioconjugation, and great blood circulation time. Figure 6 summarizes the main applications of nanogels in the biomedical field [90,92,95,107,108].

Nanogels present a great potential of use in chemotherapy, diagnosis of diseases, the release of bioactive substances and vaccines, cell culture systems, contrast agents, biocatalysis, in the generation of bioactive scaffolds in regenerative medicine, besides being able to act as sensors, nanoreactors, nanodevices, superabsorbents, and biomimetic mechanical devices, such as artificial muscles.

The interpenetrating network structure of the nanogels allows better encapsulation of drugs that can be delivered by various routes of administration such as oral, nasal, intraocular, and pulmonary pathways. Researchers working with the ocular route developed nanogels of PVP/PAA (poly(vinyl-pyrrolidone)-poly(acrylic acid) with pilocarpine allowing maintenance of the adequate concentration of the drug at the site of action for a prolonged period. Picone and collaborators developed PVP nanogels covalently attached insulin for intranasal administration demonstrating an increase in the brain insulin delivery and potential use for neurodegenerative diseases. It is also possible that nanogels entrap two drugs simultaneously, an important feature for the coadministration of two or more anticancer drugs. Most of these nanogels can be taken up by tumor cells due to their nanometer size, providing the drug delivery directly to specific sites within the cell. In this way, side effects can be minimized and bioavailability and activity can be increased [94,104,109,110].

### 4.4. Pharmaceutical Applications

Nanogels are promising and innovative materiasl in the field of pharmaceutical applications. Since the emergence of this class of materials, it has been explored for its application as biocompatible carriers with a special interest in the release of drugs due to high carrying capacity, stability, uniformity, adjustable size, ease of preparation, minimal toxicity, responsiveness to stimuli, the extensive surface area for bioconjugation, and great blood circulation time. As above-mentioned, nanogels hold suitable properties for use in chemotherapy, diagnosis of diseases, the release of bioactive substances and vaccines, and contrast agents [90,92,95,107,108].

Nanogels are used for the delivery of poorly water-soluble drugs because of their single construction formed by a hydrophobic core, micelles, and hydrophilic exterior. This conformation allows the incorporation into the core of the micelles of hydrophobic compounds and the exterior of the micellar networks the incorporation of hydrophilic compounds. Nanogels of poly (d,l-lactide-co-glycolide)-block-poly(ethylene glycol)-block-poly (d,l-lactide-co-glycolide) (PLGA-b-PEG-b-PLGA; BAB block copolymer) were developed to locally deliver hydrophobic drugs [111]. In another work [112], a thermosensitive PLGA-b-PEG-b-PLGA nanogel was developed with three poorly water-soluble compounds indicating the capacity of the nanogels of loading hydrophobic drugs despite their rich water content.

Studies involving poly(lactide)-b-PEG-b-poly (lactide) (PLA-b-PEG-b-PLA) nanogels also showed the capacity of hydrophobic drug incorporation with excellent long-term stability without aggregation. Another application of nanogels is related to the incorporation of low molecular mass drugs or biomacromolecules such as oligonucleotides, siRNA, DNA, and proteins [113]. Polyelectrolyte nanogels can incorporate them because these compounds bind with the nanogel ionic chains and phases separate within the finite nanogel volume. Therefore, the loading capacity of such nanogels is superior to most other drug carriers [111,112].

Nanogels can be used to improve the action of local anesthetics and non-steroidal anti-inflammatory drugs (NSAIDs) as well as promoting better antibacterial and antimicrobial activity. Local anesthetics block the nerve impulses in the nerve cell membrane by shutting the voltage-gated Na^+^ channels; an overdosage of them leads to their high toxicity. The incorporation of local anesthetics into nanogels can improve their regional administration. Methacrylic acid ethyl acrylate nanogel with procaine hydrochloride, a local anesthetic, showed a high release rate at high pH [114,115].

Nanogels are ideal for the development of topical delivery systems containing non-steroidal anti-inflammatory drugs (NSAIDs). These systems prepared with nanogels can overcome the limitation of brief contact time between the active drugs and the application site that topical delivery systems present. A topical delivery system of Spantid II and ketoprofen, two anti-inflammatory drugs, using nanogel of poly-(lactide-co-glycolic acid) and chitosan was developed. This system can be used in the treatment of a variety of inflammatory disorders [114,115].

Nanogel delivery systems also allow quick and localized action to treat microbial infection, a system consists of zinc nitrate, an antibacterial agent, and dextran cross-linked polyacrylamide nanogels was prepared to target the methicillin-resistant strains of *Staphylococcus aureus* using the mini-emulsion method and methacrylated hyaluronic acid as crosslinking agent [114,115].

Additionally, nanogels can be applied in cancer treatment, diabetes treatment, vaccine delivery, and for autoimmune and neurodegenerative diseases. Many studies indicate the potential of nanogel use in cancer treatment reducing the toxicity of the drug. Examples are the development of biodegradable nanogel prepared by crosslinking of polyethyleneimine and PEG/pluronic used for 5′-triphosphorylated ribavirin and crosslinked branched network of polyethyleneimine and PEG (polyplex nanogel) used for fludarabine. Oligonucleotides have shown potential use in neurodegenerative diseases such as Alzheimer’s and Parkinson’s; however, their applications in the treatment of these diseases are hindered by their instability against metabolism, their inability to penetrate the blood–brain barrier, and their rapid clearance by renal excretion. When incorporated into the nanogel delivery systems, the oligonucleotides cross the blood–brain barrier, aiding their delivery into the central nervous system [114,115].

Studies have been carried out to investigate the potential use of nanogels in bone regeneration, gastrointestinal disorder, transdermal drug delivery, autoimmune diseases, and vaccine delivery. In the process of bone regeneration, the release of lithium and other medicaments locally and slowly from biodegradable cell scaffolds is necessary. Thereby, nanogels containing lithium can be used for controlled release into bone tissue; for that purpose, nanogels were synthesized by microemulsion polymerization of polyacrylic acid and incorporated into the biodegradable polyhydroxybutyrate matriz. The nanogel application for gastrointestinal disorder was studied with the development of nanogels targeted to Human Umbilical Vein Endothelial Cells (HUVECs). Zwitterionic poly (carboxybetaine methacrylate) (p-CBMA) nanogels conjugated to cyclo [Arg-Gly-Asp-DTyr-Lys] (cRGD) were developed in a model cell system where (p-CBMA) was found to selectively bind to HUVECs [114,116].

Among the promising applications of nanogels is their use for topical delivery of active pharmaceutical ingredients to the stratum corneum. The transdermal route of administration presents benefits over other routes because it ignores the first-pass effect, promotes improvement in the drug efficiency, provides steady-state drug concentration in plasma, in addition to increasing patient compliance to treatment. Studies showed better stability and permeability of the drug aceclofenac in transdermal delivery systems using nanogels. This drug when administered orally presents side effects such as ulcers and gastric bleeding; to minimize these effects, a transdermal delivery system of the aceclofenac was developed through the emulsion solvent diffusion method. A dispersion of this drug was formed and incorporated into a gel matrix to formulate the aceclofenac nanogel.

Nanogels are also presented as an option for the treatment of autoimmune disorders; the drug delivery systems must show ability to selectively disable the immune cells that mediate the autoimmunity response. Nanogel delivery systems with immunosuppressant drugs can improve the immunosuppression effect by targeting the antigen presenting cells that contribute to disorder and allowing systemic accumulations of the drug. Nanogels prepared with the immunosuppressant mycophenolic acid (MPA) were studied for lupus erythematosus, the results indicated an increase in the patients’ survival and delay in the onset of kidney damage, a lupus complication [94,114].

Recently, a promising new generation of vaccine delivery has been obtained with the use of multiresponsive polymeric nanogels. These systems can trigger an innate immune response or enhancing antigen delivery. The intrinsic characteristics of nanogels promote advantages over conventional vaccines because the nanogel network can protect vaccine antigens from enzymatic degradation and the bioconjugation with antibodies or other ligands can increase active targeting specificity. Studies involving the development of oral vaccines have been carried out; this is cited as an example of the oral vaccine delivery system prepared with poly(2-hydroxyethyl methacrylate-*co*-methacrylic acid) P(HEMA-*co*-MAA) nanogel functionalized with mannan [92,114,115].

Nanogels have also been studied for use in diabetes treatments, gene and protein delivery, and enzymology. Diabetes presents high prevalence in the world’s population, and new treatment options are being studied as the nanogels produced with a glucose-responsive polymer that can act as self-regulated insulin delivery systems. The insulin release is dependent on the dextran content in the particles and the glucose concentration in the release medium. The hypoglycemic effect observed in these systems is equivalent to that of free insulin after administration. Proteins and peptides can be employed as therapeutic agents; however, their stabilization in delivery reservoirs at physiological pH values and temperature is a challenge as well as the proper design of carriers for sustained and targeted delivery. These limitations can be reduced with nanogel use because they reduce denaturation of proteins forming a colloidal stable complex with them on the nanometer scale.

Nowadays, among the most promising methods to diagnose and treat diseases such as cancer, neurodegenerative disorders, and viral infections is the gene therapy for the delivery of plasmid DNA (pDNA), siRNAs, micro-RNAs (miRNAs), and antisense oligodeoxynucleotides (ODNs) used in targeted inhibition of specific mRNA sequences. Nanogels have shown promising utility as non-viral carriers with low toxicity and immunogenicity and high extracellular stability and transfection efficiency [92,94,114,116].

Three-dimensional printing technology has been used to develop personalized medicines and drug delivery systems. This technology has been used to created 3D structured nanogels; some of them were 3D-printed in a structure of drug (s)/photoimitiator-loaded nanoparticles, nanoemulsions suspended in hydrogels, or liposomes. Three-dimensional-printed nanogels are also used for bioprinting in tissue engineering: acrylated Pluronic F-127 and unmodified Pluronic F-127 were mixed to create stable and strong nanostructured hydrogels via UV crosslinking. The cell viability of the encapsulated chondrocyate was increased after elution of the unmodified Pluronic F-127 from the crosslinked networks, and the mechanical strength of the nanostructured hydrogels was also increased by adding methacrylated hyaluronic acid.

Three-dimensional printing of nanogels promotes additional advantages in nanogel production, such as high precision, convenient operation, low cost, high production efficiency, and customizability. In the near future, 3D-printed nanogels carrying various therapeutic entities and biomedical materials will be widely used in regenerations of tissues/organs, wound healing, medical implants, detoxification, local disease treatments, and personalized medications. In addition, these systems will also permit rapid prototyping of soft anatomical models for preoperative surgical planning in visceral, neuro, and cardiovascular surgeries [111].

## 5. Grafted Hydrogel Chains

### 5.1. Definition

Up until this point, this work has compiled recent advances in the development of hydrogels as single entities; however, the same chemical components that may be reacted to form a hydrogel substrate may be equally covalently attached onto existing substrates to add the properties and behaviors of hydrogels to these substrates. The techniques whereby hydrogel polymer chains are attached to these substrates (also called platforms) are widely known as “grafting reactions” or “grafting procedures” and thus are useful in attaching polymer chains onto substrates ranging from polymeric films and objects to metals ceramics and composites. In a grafting procedure, necessarily, through a series of chemical modifications, it is possible to create reactive sites on a substrate so that “graft” polymer chains may be then bonded with the existing substrate. In this sense, there are several procedures available that may produce grafts onto other substrates. In many cases, it is possible not only to functionalize the surfaces of other substrates but also to attach polymeric chains on the bulk structure of a desired substrate [69].

Although these techniques have been extensively used for many polymeric systems, its use for coating of different materials with covalently attached hydrogels is very important because of the versatility of these kinds of substrates and the various available synthetic procedures that may be used to attain grafting of hydrogels onto many substrates (especially polymers). However, the choice of a specific procedure for attaining a correct graft depends heavily on the properties of both the reagents in grafting reactions, the conditions of the grafting procedure, and the desired properties of the hydrogel coating. Therefore, this section will deal with some recent examples regarding this area of application of hydrogels building upon the properties that have already been thoroughly described on this review and expanding on the possibilities regarding synthetic methods and applications of new materials obtained by these procedures.

### 5.2. Methods of Synthesis

**Figure 7:**
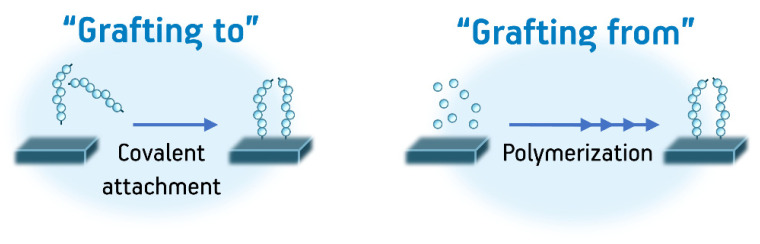
Different pathways for obtaining polymer grafts.

Grafting procedures are very well-known procedures useful for the covalent attachment of virtually any polymer onto other surfaces ranging from other polymeric materials to metal nanoparticles and carbon nanomaterials. Nevertheless, despite the advances in the field of general polymer grafts, the grafting of hydrogels is not trivial, since the grafted varieties of hydrogels must also fulfill the same structural requirements (3D crosslinked structures) that are characteristic of plain hydrogels. Therefore, often, finer synthetic control is needed to graft polymers which may act as hydrogels than for only grafting polymers. In any case, even with added difficulty, the grafting procedure of hydrogels usually follows the two main pathways that are already well-known to obtain a grafted polymeric chain onto substrates: “Grafting to” and “Grafting from”. In the first of these alternatives, preformed polymer chains may be directly attached to a surface through chemical reactions. In contrast, in the second alternative, the polymer chains are formed on the substrate by performing polymerization reactions in situ. Both procedures (Figure 7) are widely used for the formation of grafts, including grafts of hydrogel chains, and they are used to form grafts both on the surfaces and on the bulk microstructures of a substrate [69,117].

The most substantial difference when grafting hydrogels onto other substrates when comparing with typical grafting of polymers is the cross-linking needed to form the 3D structures intrinsic of hydrogels. Additionally, many procedures need chemical spacers between the substrates and the polymer chain to achieve better polymerizations on the surface of the material, control the distribution of chains on the substrate, or even control the architecture of the final hydrogel coating. Due to the variety of the current methods that fulfill the aforementioned synthetic requirements, a classification of these methods according to their reaction mechanisms is helpful when deciding how to obtain a specific hydrogel. This classification is listed below [4,39,69]:

- Grafting by radical polymerizations: This method is among the most common as it is relatively easy to apply to a great variety of surfaces and it works on virtually any monomer containing vinyl groups. In these procedures, typically, functional groups active on radical polymerization (vinyl groups, azo groups, peroxides, i.e.,) are immobilized through chemical means on the substrate that will be grafted. Subsequently, a radical polymerization reaction is performed in the presence of a crosslinker (or even the absence of a crosslinker depending on the nature of the monomer). As mentioned before, these reactions are the most versatile in terms of applicability to different substrates. Nevertheless, these kinds of reactions provide little to no synthetic control over the finer structure of the hydrogel since it is impossible to control polymeric chain length and polymer dispersity; additionally, it is not possible to achieve the formation of specific polymeric architectures (block copolymers, star copolymers, dendrimers, etc.) with these procedures. This in turn affects how useful the final hydrogel product is. Another disadvantage of this method is the presence of residual toxic initiators and crosslinkers on the final product, which limit the application of these kinds of grated materials in biomedicine [117,118].
- Grafting by controlled radical polymerizations: Controlled radical polymerizations include procedures such as atom-transfer radical polymerization (ATRP), reverse addition-fragmentation chain-transfer (RAFT) polymerization, ring-opening metathesis polymerization (ROMP), and nitroxide-mediated radical polymerization (NMP). These procedures are also widely used for the formation of grafts of hydrogels because of their strong versatility in producing exotic architectures that may be very useful when crafting complicated aerogels. These procedures use special initiators coupled with catalysts that allow for polymerization with very controlled molecular weight distributions, control over the molecular weights of the polymers, and overall control on all the structural parameters of the polymer chains. Although these procedures are very useful, their use is limited by the elevated cost of the initiator-catalysts systems and the synthetic challenge of producing monomers (and substrates to graft) that are compatible with some of these procedures. Additionally, the issue with toxic residues on the products still limits the applications of these polymers in some areas such as the biomedical fields [119,120].
- Grafting polymerizations induced by radiation: This final classification for the grafting of hydrogels is very important since it tends to overcome the problem of residual toxic polymerization initiators, crosslinkers, and spacers. In these procedures, reactive monomers are activated by high energy radiation, ranging from UV to gamma radiation. The latter even being able to form cross-linkages without the use of chemical additives as it has been mentioned for plain hydrogels. These materials have then had many applications, including several the biomedical field [69].

### 5.3. Biomedical Applications

Grafted materials, especially polymers, have an enormous amount of applications in biomedical fields because many polymeric substrates are already highly important in medical devices such as urinary and central line catheters, prosthetics, wound dressings, etc. Therefore, the modification of these devices with hydrogels for the production of functional materials have been widely exploited. For instance, grafted hydrogels may be used for the development of substrates with modified hydrophilicity and swelling characteristics [121,122,123], antimicrobial and antifouling surfaces [124,125,126], scaffolds for tissue engineering [127,128], smart materials [129,130], and localized drug delivery [131].

A recent example of one of these applications is the development of hydrogels grafted onto titanium surfaces for the development of prosthetic materials with increased biocompatibility and improved mechanical properties upon adhesion to bone and teeth. In this work, the authors first introduced SiO_2_ onto the surface of the metals by using a grit-blasting procedure, later introducing methacrylate groups which bonded through silane bonds to the surface of the titanium and were able to react with monomers such as poly(ethylene glycol) dimethacrylate (PEGDMA) coupled with PHEMA, and poly(ethylene glycol) diacrylate (PEGDA) coupled with PAA both onto simple networks and interpenetrated networks. All of these polymerizations were performed by UV activation. In this work, it was possible to obtain reproducible bonding of adhesive hydrogels to polymer surfaces for better application in orthopedics [132].

Another interesting example of these applications was the work performed by Chao and collaborators in 2018. In this work, the authors used a procedure to modify polydimethylsiloxane (PDMS) catheters by using a variety of surface initiated ATRP (SARA SI-ATRP: supplemental activator and reducing agent surface-initiated atom transfer radical polymerization) to form positively charged hydrogels of diallyl dimethyl ammonium chloride (DADMAC), or ε-poly-L-lysine HCl methacrylic acid (EPL-MA) crosslinked with PEGDMA. This grafted hydrogels demonstrated good antibacterial activity against methicillin-resistant *Staphylococcus aureus* and vancomycin-resistant *Enterococcus* strains [133]. In another recent example, Velazco-Medel and collaborators grafted acrylic acid with PEGDMA as a crosslinker and as a comonomer by using gamma radiation as polymerization initiation. In this study, the authors studied the effect of radiation dose (input energy to the system), the ratio between the reagent and their concentration, on the effectiveness of the graft; additionally, as expected, these materials had improved hydrophilicity and pH-responsiveness observable both in swelling studies and acid-base titrations. The properties of these materials suggest possible uses for drug delivery applications and antibiofilm uses in medical devices [134].

### 5.4. Pharmaceutical Applications

Pharmaceutical applications of grafted hydrogels are also very interesting because of the versatility of the materials that may be produced by these techniques. However, specific examples of grafted hydrogels that are useful as pharmaceutical forms are not so common, since plain hydrogels are already very useful as pharmaceutical forms (see previous sections). Even so, some examples of these kinds of systems have been produced in recent years. In this section, we will present a brief compilation of some interesting examples.

On the first of these examples, Peppas et al. compiled the advances of their research of PEG-grafted poly(methyl methacrylate) for drug delivery in oral formulations within a review of hydrogels as oral formulation. Their group performed this series of studies by modulating the pH properties, metabolism characteristics of the hydrogels, and potential for drug delivery. This review is left to the reader as it is an excellent compilation of several hydrogels crafted for oral formulations [34].

Another application of grafted hydrogels is injectable formulations for drug delivery. As a first example of these applications, a polyurethane hydrogel was grafted onto chitosan using a “grafting to” procedure. To achieve this synthesis, the authors formed a prepolymer through a polycondensation, yielding a polyurethane containing isocyanate end groups. This prepolymer was later reacted with chitosan using dibutyltin dilaurate as a catalyst. In this study, hydrophilicity, mechanical properties, swelling capability, biodegradability, and the ability to encapsulate drugs were studied. It was found that these materials are capable of acting as injectable substrates while delivering drug substrates in a controlled manner [135]. As another example of injectable hydrogel systems, polyethylene grafted chitosan materials have been produced through EDC carbonyl reactions in order to obtain a hydrogel grafted system that may react to temperature and pH. In this work, they were able to modulate the sol-gel transition temperature and pH values of the systems to correspond to the conditions of acidic tumors. It was found that these systems may release doxorubicin and curcumin for a sustained period of up to two weeks [136]. As a final example, hydrogels have also been grafted onto alginate-based polymers; for instance, in a 2019 research paper by Iatridi and collaborators, a PNIPAM and N-*tert*-butylacrylamide were researched for the temperature dependence of their sol-gel transitions [137].

## 6. Conclusions

In this review, we aimed to produce an updated overview of the recently published studies concerning methods of synthesis, and biomedical and pharmaceutical applications of macro-, micro-, nano-, and grafted hydrogels. Although hydrogels are also of interest to other fields such as agriculture and environment protection, their potentialities in biomedical and pharmaceutical research are the most promising. That is strictly related to their good biocompatibility and capacity of absorbing great amounts of water and maintaining shape and mechanical properties.

The possibility of molding the hydrogel according to the needs of the application, selecting the appropriated structures, molecules, pore sizes, and responsiveness to specific stimuli is what makes these materials such promising strategies for solving pharmaceutical and biomedical issues. These possibilities, however, may increase the complexity of the systems and required different methodologies for proper assessment of their properties.

Micro- and nanogels have lower viscosity, higher surface area, and respond very fast to stimuli from the environment, which in turn makes them very suitable to load drugs, especially sensitive and rapidly metabolized ones. Nanogels, more specifically, present high carrying capacity and great blood circulation time, beyond the ability to deliver the active locally and intracellularly. All of these features are prodigious to the enhancement of the efficiency of treatments such as chemotherapies that are still limited and full of side effects.

Thus, hydrogels may be the solution for several limitations and unsolved problems in the medical and pharmaceutical areas, representing the evolution of already existing treatments and new possibilities of approaches. Although they are already known as the materials of the XXI century, much more is yet to come, such as improved biopharmaceutical advantages, controlled drug release, stimuli-responsiveness for remote signals, and new designs to explore different routes of administration, for instance.

## Figures and Tables

**Table 1:** An overview of the processes for microgel formation.

Method		Brief Description
Homogeneous Nucleation	Emulsion Polymerization	Water-soluble monomers and crosslink agents are mixed in an aqueous medium. In this case, initially, a homogeneous solution is obtained. To avoid the formation of a macrogel, it is of great importance that the polymer formed is not soluble under the conditions for polymerization [63].
Emulsification: W/O Heterogeneous Emulsion	Emulsion Polymerization	Water-soluble monomers and bioactive components are dispersed in an oil medium with surfactants and high shear forces, forming a colloidal system. To promote particle gelation inside the water droplets, several methods may be used such as chemical crosslink agents or stimulating it by temperature according to the polymer critical temperature [65,66].
	Inverse Microemulsion Polymerization	Monomer aqueous droplets are dispersed in an oil phase by a homogenizer or mechanical stirrer. Inside these aqueous droplets, drugs and other substances of interest may be incorporated. Crosslinking agents are used for this process [59].
	Membrane Emulsification	In this process, an emulsion is passed through the pores of a membrane made of glass or ceramic into a nanofluid phase to form a microgel [61].
	Heterogeneous controlled/living radical polymerization	This process may be performed by several methods such as stable radical polymerization, reversible addition-fragmentation chain transfer, and transfer radical polymerization [61].
Polymer Complexation		Microgels are obtained by mixing polymeric solutions of opposite charges, forming polyelectrolyte complexes [67].
Radiation		To produce microgels by radiation, the polymer solution is placed in molds, which may be the final packages, then they are exposed to γ-rays that will crosslink and sterilize the microgels. In this case, crosslinking occurs from the water radiolysis that generates hydroxyl radicals that react with polymer chains allowing them to combine. Radiation may also be used to make polymer surface modifications and obtain physical-chemical properties of interest [68,69].
Physical-Based Methods for Microgel Fabrication	Photolithographic Techniques	In this method, a monomer solution containing a photoinitiator, and the crosslinking agent is exposed to ultraviolet or laser light which causes the curing reaction. Masks and stamps are used to control the size and shape of the microgel [59].
	Micromolding Method	This method is like photoligraphy, but in this case, the polymer solution is placed in a mold, and gelation happens by temperature change or by adding a gelling agent [64].
	Microfluidic and Droplet Formation	It combines the synthesis of polymer particles and microencapsulation. In this case, polymer solutions are injected in an oil phase and then crosslinked. The difference here is the use of specially designed devices that allow specific morphologies and structures for the particles formed [65].

**Table 2:** Methods of nanogel synthesis.

Traditional Method	Simultaneous polymerization and crosslinking	Emulsion polymerization [92,103]
		Precipitation polymerization [92]
		Inverse emulsion polymerization [92,103]
		Dispersion polymerization [92]
		Controlled radical polymerization [105]
		Atom transfer radical polymerization (ATRP) [92,103]
		Reversible addition-fragmentation chain transfer (RAFT) polymerization [92]
		Degenerative chain transfer polymerization represented by iodine-mediated polymerization [106]
		Uncontrolled radical polymerization [92]
	Crosslinking of polymer precursors	Disulfide-based crosslinking [92,103,105]
		Amine-based crosslinking [92,103,105]
		Imine crosslinking [92,105]
		Click chemistry-based crosslinking [92,103]
		Photoinduced crosslinking [92,103,105]
		Physical crosslinkingc [92,103,105]
	Controlled aggregation by physical self-assembly of hydrophilic polymers [90]	
	Template-assisted fabrication of nanogel particles	Photolithography [90,92,94]
		Micromolding techniques [90,92,94]
Novel Methods	Novel pullulan chemistry modification [94]	
	Novel photochemical approach [94]	
	Novel radical polymerization with inverse mini-emulsion technology [94]	
	Addition-fragmentation transfer process [94]	
	Chemical modification [94]	
